# Coronavirus Pandemic - #STAYHOME: How Are You Holding Up? Questions And Tips For 11-18 Year Olds To Make It Better

**DOI:** 10.1192/j.eurpsy.2022.783

**Published:** 2022-09-01

**Authors:** D. Szentivanyi, L.O. Horváth, K. Buist, B. Farkas, G. Dallos, P. Garas, D. Győri, J. Balazs

**Affiliations:** 1 Eötvös Loránd University, Institute Of Psychology, Izabella, Hungary; 2 Eötvös Loránd University, Institute Of Psychology, Department Of Developmental And Clinical Child Psychology, Budapest, Hungary; 3 Utrecht University, Utrecht University, Utrecht, Netherlands; 4 Semmelweis University, Doctoral School Of Mental Health Sciences, Budapest, Hungary; 5 Semmelweis University, Mental Health Sciences School Of Ph.d. Studies, Budapest, Hungary; 6 Eötvös Loránd University, Doctoral School Of Psychology, Budapest, Hungary; 7 Eotvos Lorant University, Developmental And Clinical Child Psychology, Budapest, Hungary

**Keywords:** COVID-19 pandemic, restrictions, adolescent, mental health, quality of life, online prevention progr

## Abstract

**Introduction:**

Adolescents have to cope with several challenges and restrictions due to the COVID-19 pandemic, with many of those incongruent with the typical developmental tasks of adolescent age. Some adolescent might be particularly vulnerable in this situation.

**Objectives:**

This study aimed: 1) to collect data on the mental health and quality of life of adolescents during/after the pandemic; 2. to improve adolescents’ mental health by providing an online prevention program that addresses their actual needs; 3. to accelerate the development of culturally adapted prevention programs by involving an international team, and 4. to contribute to adequate preparation for any similar situation in the future.

**Methods:**

Participants aged 11-18 years and their parents/caregivers were recruited online. Data has been collected regularly in a follow-up study by Inventar zur Erfassung der Lebensqualitat and Strengths and Difficulties Questionnaire. The baseline data collection was in March 2020 at first restrictions of the COVID pandemic in Europe

**Results:**

In the baseline data 428 adolescents (29.7% boys;70.3% girls) were included. Adolescents reported significantly lower quality of life during the pandemic (F (1,557) = 29.11; p <0.001; R2 = 0.048). There was no significant difference in quality of life according to whether the adolescents live in a household with their siblings ( F (2, 356) = 0.785 p = 0.457; η2 = 0.004), and whether the adolescents have symptoms of hyperactivity (ß = 0.105; p = 0.295).

**Conclusions:**

Prevention based on the results of this study is expected to contribute to maintaining adolescents’ mental health during and after the COVID pandemic.

**Disclosure:**

No significant relationships.

